# Supplementary material for: Mitogen-Activated Protein Kinases Associated Sites of Tobacco Repression of Shoot Growth Regulates Its Localization in Plant Cells
Source: Int J Mol Sci. 2022 Aug 11;23(16):8941. doi: 10.3390/ijms23168941 (PMC9409217; doi:10.3390/ijms23168941)
Supplement: Supplementary file 1 [file ijms-23-08941-s001.zip › Supplementary Figures.pdf]

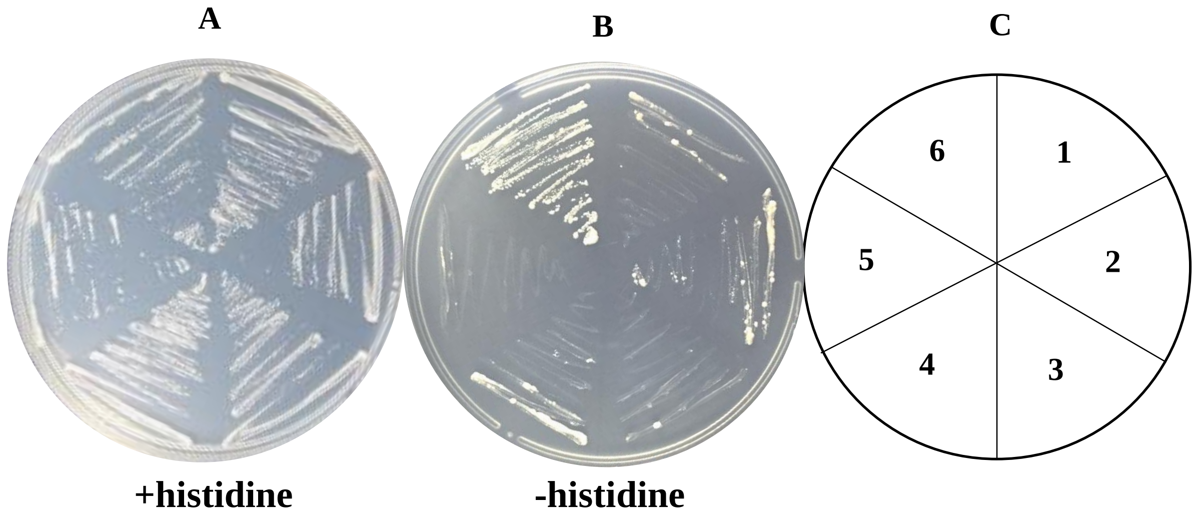

Figure S1. Yeast-two-hybrid assay between 3 potential substrate binding domains of NtMPK3 and NtRSG. The indicated cell cultures were plated and grown on non-selective (+histidine) (A) and selective (–histidine) (B). Yeast-two-hybrid assay of tested protein pairs are arranged as indicated in (C) accordingly. 1: pSTT91-NtMAPK3 (Acitve site 48-246aa), pGAD424-TMV-MP. 2: pSTT91-NtMAPK3 (Acitve site 48-246aa), pGAD424-RSG ; 3: pSTT91-NtMAPK3 (Polypeptide substrate binding site 90-251aa), pGAD424-TMV-MP. 4: pSTT91-NtMAPK3 (Polypeptide substrate binding site 90-251aa), pGAD424-RSG. 5: pSTT91-NtMAPK3 (KIM domain 91-342aa), pGAD424-TMV-MP. 6: pSTT91-NtMAPK3 (KIM domain 91-342aa), pGAD424-RSG.

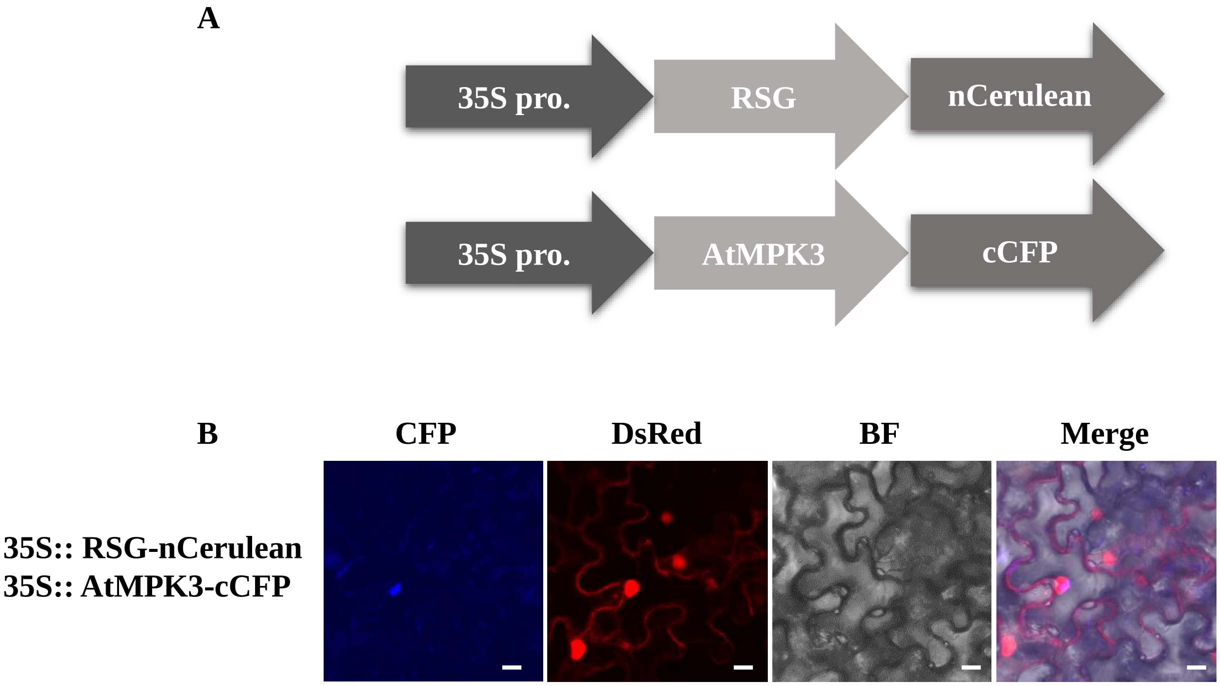

Figure S2. RSG interacts with Arabidopsis mitogen-Activated Protein Kinase 3 (MAPK3). (A) Constructs of 35S-driven RSG-nCerulean and 35S-driven AtMPK3-cCFP. (B) BiFC assay. RSG-nCerulean and AtMPK3-cCFP were transiently expressed in agroinfiltrated leaf epidermis of *N. benthamiana*. NLS-VirD2 fused with DsRed indicated the localization of nuclear. and analyzed by confocal microscopy three days post-infiltration. CFP signal is in cyan. Images are single confocal sections, representative of images obtained in two independent experiments performed for each protein; for each experiment, three infiltrations were performed on three different leaves, with two images recorded per infiltration. Scale bars= 20 μM.
