# Supplementary material for: Mitogen-Activated Protein Kinases Associated Sites of Tobacco Repression of Shoot Growth Regulates Its Localization in Plant Cells
Source: Int J Mol Sci. 2022 Aug 11;23(16):8941. doi: 10.3390/ijms23168941 (PMC9409217; doi:10.3390/ijms23168941)
Supplement: Supplementary file 1 [file ijms-23-08941-s001.zip › Table S1-2.pdf]

Table S1. Plasmids and cloning strategies used in this study.

Plasmid or bacterial strain	Description	Source or reference	Primers used
pGAD424	Plasmid carrying Gal4 activation domain to clone libraries or known genes for interactions with bait; Amp^r, LEU1+ (Clontech)	Clontech/Matchmaker	
pGAD424-RSG	RSG (from *N. tobacco*) cDNA sequence subcloned as MfeI/SalI fragment into the multiple cloning site of pGAD424	This study	1F/1R
pGAD424-RSG-T28A	Two PCR products obtained with the primer pairs 1F-11R and 11F-1R with pGAD424-RSG as template were used as template with primers 1F-1R to generate RSG-T28A, introduced as MfeI/SalI into pGAD424	This study	11F/11R<br>1F/1R
pGAD424-RSG-T30A	Two PCR products obtained with the primer pairs 1F-12R and 12F-1R with pGAD424-RSG as template were used as template with primers 1F-1R to generate RSG-T28A, introduced as MfeI/SalI into pGAD424	This study	12F/12R<br>1F/1R
pGAD424-RSG-S74A	Two PCR products obtained with the primer pairs 1F-13R and 13F-1R with pGAD424-RSG as template were used as template with primers 1F-1R to generate RSG-T28A, introduced as MfeI/SalI into pGAD424	This study	13F/13R<br>1F/1R
pGAD424-RSG-T135A	Two PCR products obtained with the primer pairs 1F-14R and 14F-1R with pGAD424-RSG as template were used as template with primers 1F-1R to generate RSG-T28A, introduced as MfeI/SalI into pGAD424	This study	14F/14R<br>1F/1R

pGAD424-TMV-MP	full-length TMV MP (encoding the movement protein of *Tobacco mosaic virus*, JX993906.1) cDNA cloned as BamHI-PstI PCR fragment into pGAD424 ()	Lab stock (Vitaly Citovsky)	
pSTT91	Yeast expression vector, carrying LexA DNA binding domain for fusion with prey proteins (derived from pBTM116); Amp^r, TRP1+ (Hollenberg, 1995)	Stefan Tafrov	
pSTT91-NtMPK3	MPK3 (XP_016478177.1 from *N. tobacco*) cDNA sequence subcloned as EcoR1/BamHI fragment into the multiple cloning site of pSTT91	This study	2F/2R
pSTT91-NtMPK3-AT	Active site (48-246aa) of NtMPK3 was subcloned as EcoR1/BamHI fragment into the multiple cloning site of pSTT91	This study	3F/3R
pSTT91-NtMPK3-PSB	Polypeptide substrate binding site (90-251aa) of NtMPK3 EcoR1/BamHI fragment into the multiple cloning site of pSTT91	This study	4F/4R
pSTT91-NtMPK3-KD	KIM domain site (91-342aa) of NtMPK3 EcoR1/BamHI fragment into the multiple cloning site of pSTT91	This study	5F/5R
pSAT1-nCerulean-C1	Plant expression vector for fusions with nCerulean, driven by 35S promoter expressed in plants (Tzfira *et al*., 2005)	Lab stock (Vitaly Citovsky)	
pSAT1-nCerulean-RSG	RSG (from *N. tobacco*) cDNA sequence subcloned as EcoR1/BamHI fragment into the multiple cloning site of pSAT1-nCerulean-C1	This study	6F/6R
pSAT1-nCerulean-RSG-T28A	pGAD424-RSG-T28A as template were used as template with primers 6F-6R to generate RSG-T28A, introduced as EcoR1/BamHI into pSAT1-nCerulean-C1	This study	6F/6R
pSAT1-nCerulean-RSG-T30A	pGAD424-RSG-T30A as template were used as template	This study	6F/6R

	with primers 6F-6R to generate RSG-T30A, introduced as EcoR1/BamHI into pSAT1-nCerulean-C1		
pSAT1-nCerulean-RSG-S74A	pGAD424-RSG-S74A as template were used as template with primers 6F-6R to generate RSG-S74A, introduced as EcoR1/BamHI into pSAT1-nCerulean-C1	This study	6F/6R
pSAT1-nCerulean-RSG-T135A	pGAD424-RSG-T135A as template were used as template with primers 6F-6R to generate RSG-T135A, introduced as EcoR1/BamHI into pSAT1-nCerulean-C1	This study	6F/6R
pPZP-RCS2	Binary vector carrying homing endonuclease sites in its T-DNA, allowing the introduction of expression cassettes from satellites (pSAT series) plasmids (Tzfira *et al*., 2005), $Spec^r$	Lab stock (Vitaly Citovsky)	
RCS2-nCerulean-RSG	nCerulean-RSG expression cassette as AscI fragment from pSAT1-nCerulean-RSG into pPZP-RCS2	This study	
RCS2-nCerulean-RSG-T28A	nCerulean-RSG-T28A expression cassette as AscI fragment from pSAT1-nCerulean-RSG-T28A into pPZP-RCS2	This study	
RCS2-nCerulean-RSG-T30A	nCerulean-RSG-T30A expression cassette as AscI fragment from pSAT1-nCerulean-RSG-T30A into pPZP-RCS2	This study	
RCS2-nCerulean-RSG-S74A	nCerulean-RSG-S74A expression cassette as AscI fragment from pSAT1-nCerulean-RSG-S74A into pPZP-RCS2	This study	
RCS2-nCerulean-RSG-T135A	nCerulean-RSG-T135A expression cassette as AscI fragment from pSAT1-nCerulean-RSG-T135A into pPZP-RCS2	This study	
pSAT5-cCFP-C1	Plant expression vector for fusions with cCFP, driven by 35S promoter expressed in plants (Tzfira *et al*., 2005)	Lab stock (Vitaly Citovsky)	
pSAT5-cCFP-NtMPK3	MPK3 (XP_016478177.1 from *N. tobacco*) cDNA sequence subcloned as EcoR1/SalI fragment into the multiple cloning	This study	7F/7R

	site of pSAT5-cCFP-C1		
pSAT5-cCFP-AtMPK3	MPK3 (OAP03702.1 from *A. thaliana*) cDNA sequence subcloned as EcoR1/SalI fragment into the multiple cloning site of pSAT5-cCFP-C1	This study	8F/8R
pPZP-RCS2-NLS	Binary vector carrying homing endonuclease sites in its T-DNA, for stable expression of nucleus mRFP-VirD2NLS marker in plants; mRFP-VirD2NLS expression cassette cloned as PIPspI fragment into the same site of RCS2	Lab stock (Vitaly Citovsky)	
RCS2-NLS-cCFP-NtMPK3	cCFP-NtMPK3 expression cassette as IceuI fragment from pSAT5-cCFP-NtMPK3 into pPZP-RCS2-NLS	This study	
RCS2-NLS-cCFP-AtMPK3	cCFP-AtMPK3 expression cassette as IceuI fragment from pSAT5-cCFP-AtMPK3 into pPZP-RCS2-NLS	This study	
pSAT5-CFP-N1	Plant expression vector for fusions with CFP, driven by 35S promoter expressed in plants; Amp^r (Tzfira *et al*., 2005)	Lab stock (Vitaly Citovsky)	
pSAT5 -RSG-CFP	RSG (from *N. tobacco*) cDNA sequence subcloned as EcoR1/BamHI fragment into the multiple cloning site of pSAT1-CFP-N1	This study	9F/9R
pSAT5 -RSG-T28A-CFP	pGAD424-RSG-T28A as template were used as template with primers 9F-9R to generate RSG-T28A, introduced as EcoR1/BamHI into pSAT1-CFP-N1	This study	9F/9R
pSAT5 -RSG-T30A-CFP	pGAD424-RSG-T30A as template were used as template with primers 9F-9R to generate RSG-T30A, introduced as EcoR1/BamHI into pSAT1-CFP-N1	This study	9F/9R
pSAT5 -RSG-S74A-CFP	pGAD424-RSG-S74A as template were used as template with primers 9F-9R to generate RSG-S74A, introduced as EcoR1/BamHI into pSAT1-CFP-N1	This study	9F/9R

pSAT5 -RSG-T135A-CFP	pGAD424-RSG-T135A as template were used as template with primers 9F-9R to generate RSG-T135A, introduced as EcoR1/BamHI into pSAT1-CFP-N1	This study	9F/9R
pSAT5 -RSG-3M-CFP	Fragment of RSG-3M was generated with 3-steps fusion with pSAT5-CFP-RSG as template, primers 15F-15R for mutation in T28D, primers 16F-16R for mutation in S74D and primers 17F-17R for mutation in T135D, RSG-3M was introduced as EcoR1/BamHI into pSAT1-CFP-N1	This study	15F/15R<br>16F/16R<br>17F/17R<br>9F/9R
pPZP-RCS2-nptII	binary pPZP-RCS2 vector expressing kanamycin resistance gene nptII under NOS promoter and terminator	Lab stock (Vitaly Citovsky)	
RCS2-RSG-CFP	RSG-CFP expression cassette as IceuI fragment from pSAT5-RSG-CFP into RCS2-nptII	This study	
RCS2-RSG -T28A-CFP	RSG-T28A-CFP expression cassette as IceuI fragment from pSAT5-RSG-T28A-CFP into RCS2-nptII	This study	
RCS2-RSG -T30A-CFP	RSG-T30A-CFP expression cassette as IceuI fragment from pSAT5-RSG-T30A-CFP into RCS2-nptII	This study	
RCS2-RSG -S74A-CFP	RSG-S74A-CFP expression cassette as IceuI fragment from pSAT5-RSG-S74A-CFP into RCS2-nptII	This study	
RCS2-RSG -T135A-CFP	RSG-T135A-CFP expression cassette as IceuI fragment from pSAT5-RSG-T135A-CFP into RCS2-nptII	This study	
RCS2-RSG-3M	RSG-3M-CFP expression cassette as IceuI fragment from pSAT5-RSG-3M-CFP into RCS2-nptII	This study	
pSAT5-MYC-N1	Plant expression vector for fusions with N-terminal MYC tag, expression cassette can be transferred into I-CeuI of pPZP-RCS2; Amp^r (Magori et al., 2011)	Lab stock (Vitaly Citovsky)	
pSAT5-NtMPK3-MYC	MPK3 (XP_016478177.1 from *N. tobacco*) cDNA sequence	This study	10F/10R

	subcloned as EcoR1/BamHI fragment into the multiple cloning site of pSAT5-MYC-N1		
RCS2-NtMPK3-MYC	NtMPK3-MYC expression cassette as IceuI fragment from pSAT5-NtMPK3-MYC into pPZP-RCS2	This study	
Strain			
DH5α	*Escherichia coli*	Lab stock (Vitaly Citovsky)	
EHA105	disarmed, super-virulent *Agrobacterium* strain; Rif^r	Lab stock (Vitaly Citovsky)	
Yeast			
TAT7	*Saccharomyces cerevisiae* strain TAT7 (L40-ura3)	Lab stock (Vitaly Citovsky)	

Table S2. Primer sequences shown in this study.

	Name	Sequence
1F	RSG-MfeI-f	CCGCAATTGTGGATCCCACTCCACCCC
1R	RSG-SalI-r	ACGCGTCGACCATAAAAGATGTTGAACAATATCTAGCT
2F	NtMPK3-EcoRI-f	CCGGAATTCATGGCTGATGCAAATATGGG
2R	NtMPK3-BamHI-r	CGCGGATCCTTAAGCATATTCAGGATTCAG
3F	NtMPK3AT-EcoRI-f	CCGGAATTCCCTATTGGTCGTGGTGCTTAT
3R	NtMPK3AT-BamHI-r	CGCGGATCCTTATTTTCCAGCAAACAAAGGTTT
4F	NtMPK3PSB-EcoRI-f	CCGGAATTCACTCTCCGTGAGATTAAGCTC
4R	NtMPK3PSB-BamHI-r	CGCGGATCCTTAATCTTTTCCAGCAAACAAAGG
5F	NtMPK3KD-EcoRI-f	CCGGAATTCCATTTAGACCATGAAAATGTA
5R	NtMPK3KD-BamHI-r	CGCGGATCCTTACGGTTCGTCACCTGCATCGTG
6F	RSG-EcoRI-f	CCGGAATTCTATGGACCCGAAGTTCAGCGG
6R	RSG-BamHI-r	CGCGGATCCTCAACCCCTGTTATTGAAGTTCATG
7F	NtMPK3-EcoRI-f	CCGGAATTCTATGGCTGATGCAAATATGGG
7R	NtMPK3-SalI-r	ACGCGTCGACTTAAGCATATTCAGGATTCAGTG
8F	AtMPK3-EcoRI-f	CCGGAATTCTATGAACACCGGCGGTGGCCAATA
8R	AtMPK3-SalI-r	ACGCGTCGACCTAACCGTATGTTGGATTGAGTG
9F	RSG-EcoRI-f	CCGGAATTCATGGACCCGAAGTTCAGCGGAAAG
9R	RSG-BamHI-r	CGCGGATCCCACCCCTGTTATTGAAGTTCATGAAG
10F	NtMPK3-EcoRI-f	CCGGAATTCATGGCTGATGCAAATATGGG
10R	NtMPK3-BamHI-r	GCGGGATCCCAGCATATTCAGGATTC
11F	RSG-T28A-f	ATGCCGGATGCTCCGACCCGTATAG
11R	RSG-T28A-r	TATACGGGTCGGAGCATCCGGCATC
12F	RSG-T30A-f	ATACTCCGGCCCGTATAGCACGT

12R	RSG-T30A-r	ACGTGCTATACGGGCCGGAGTAT
13F	RSG-S74A-f	CGTCGCTAGCCCCTTCTGCTGATACT
13R	RSG-S74A-r	AGAAGGGGCTAGCGACGGCGCTGAAATA
14F	RSG-T135A-f	ATGGTTTGGAGTTTGGAGCGACCGCG
14R	RSG-T135A-r	CGGCGCGGTCGCTCCAAACTCCAAACCAT
15F	RSG-T30D-f	ATACTCCGGACCGTATAGCACGT
15R	RSG-T30D-r	ACGTGCTATACGGTCCGGAGTAT
16F	RSG-T74D-f	CGTCGCTAGACCCTTCTGCTGATACT
16R	RSG-T74D-r	AGTATCAGCAGAAGGGTCTAGCGACG
17F	RSG-T135D-f	ATGGTTTGGAGTTTGGAGCGACCGACCCG
17R	RSG-T135D-r	CGGGTCGGTCGCTCCAAACTCCAAACCAT
18F	NtGA20ox-RT-f	CAACGCCCATCGTTTCATGG
18R	NtGA20ox-RT-r	CAAAAACTTGAAGCCCGCCA
19F	NtarcA-RT-f	ATGTGTTCGTTTCAGCCCGA
19R	NtarcA-RT-r	CCGCTGAAAAGTGTGCTTCC
